# Impact of Epstein–Barr Virus Nuclear Antigen 1 on Neuroinflammation in PARK2 Knockout Mice

**DOI:** 10.3390/ijms251910697

**Published:** 2024-10-04

**Authors:** Davide Cossu, Yuji Tomizawa, Sachiko Noda, Eiichi Momotani, Tamami Sakanishi, Hanna Okada, Kazumasa Yokoyama, Leonardo Antonio Sechi, Nobutaka Hattori

**Affiliations:** 1 Department of Neurology, Juntendo University, Tokyo 1138431, Japan; zawa@juntendo.ac.jp (Y.T.); s-noda@juntendo.ac.jp (S.N.); ha-okada@juntendo.ac.jp (H.O.); kazumasa@juntendo.ac.jp (K.Y.); nhattori@juntendo.ac.jp (N.H.); 2 Biomedical Research Core Facilities, Juntendo University, Tokyo 1138431, Japan; 3 Department of Biomedical Sciences, Sassari University, 07100 Sassari, Italy; sechila@uniss.it; 4 Comparative Medical Research Institute, Tsukuba 305-0856, Japan; eiichimomotani@gmail.com; 5 Division of Cell Biology, Juntendo University, Tokyo 1138431, Japan; tama@juntendo.ac.jp; 6 Complex Structure of Microbiology and Virology, University Hospital, 07100 Sassari, Italy; 7 Neurodegenerative Disorders Collaborative Laboratory, RIKEN Center for Brain Science, Saitama 3510918, Japan

**Keywords:** Epstein–Barr virus, PARK2 knockout mice, mitochondrial dysfunction, experimental autoimmune encephalomyelitis, neuroinflammation

## Abstract

This study aimed to explore the intricate relationship between mitochondrial dysfunction, infection, and neuroinflammation, focusing specifically on the impact of pathogenic epitopes of the Epstein–Barr Virus (EBV) nuclear antigen 1 (EBNA1) in a mouse model of mitochondrial dysfunctions. The investigation included female middle-aged *PARK2^−/−^* and C57BL/6J wild-type mice immunized with EBNA1_386–405_ or with active experimental autoimmune encephalomyelitis (EAE) induction by the myelin oligodendrocyte glycoprotein (MOG)_35–55_ peptide. The *PARK2^−/−^* mice developed more severe EAE than the wild-type mice. Following immunization with EBNA1_386–405_, only *PARK2^−/−^* exhibited symptoms resembling EAE. During the acute phase, *PARK2^−/−^* mice immunized with either MOG_35–55_ or EBNA1_386–405_ exhibited a similar infiltration of the T cells and macrophages in the spinal cord and decreased glial fibrillary acidic protein (GFAP) expression in the brain. However, the EBNA1_386–405_ -immunized *PARK2^−/−^* mice showed significantly increased frequencies of CD8a^+^ T cells and CD11c^+^ B cells, and distinct cytokine profiles in the periphery compared to the wild-type controls. These findings highlight the role of EBV in exacerbating inflammation, particularly in the context of mitochondrial deficiencies.

## 1. Introduction

The exact cause of autoimmune responses in neuroinflammatory disorders such as multiple sclerosis (MS) remains unclear. While certain genetic variations increase the risk, they do not guarantee disease onset. Environmental factors, including infections, significantly contribute to the development of central nervous system (CNS) inflammatory disorders such as MS [1]. However, direct evidence of a specific pathogenic agent causing MS is still lacking.

The Epstein–Barr virus (EBV), a member of the herpes virus family which establishes latent infection with periodic reactivation, is considered a potential environmental trigger in MS development, possibly by infecting B cells [2]. Research has shown a correlation between prior EBV infection and an increased risk of MS onset [3]. This suggests that molecular mimicry, where EBV antigens resemble self-antigens, may induce autoimmunity in genetically susceptible individuals [4]. Given that over 90% of the global population is seropositive for EBV [5], other factors such as interactions with additional pathogens, vaccination profiles, and specific genetic variations likely contribute to the difference in MS prevalence between high- and low-risk areas. These factors can also influence the highly heterogeneous disease course.

Mitochondrial dysfunction plays an important role in the progression of neurological disorders, including MS, contributing to energy deficiency, oxidative stress, inflammation, and neurodegeneration [6]. Reduced ATP production due to mitochondrial dysfunctions can weaken the immune system, causing metabolic failure in peripheral immune cells [7]. In MS, demyelinated axons require more energy to propagate action potentials, and mitochondrial dysfunction exacerbates the energy deficit, further compromising neuronal function.

Infections can impact mitochondrial function and, conversely, mitochondrial dysfunction can influence the body’s ability to respond to infections [8]. Infections can lead to the increased production of reactive oxygen species and the release of cytokines, disrupting mitochondrial dynamics [9]. This bidirectional relationship between mitochondrial dysfunction and infections highlights the complex interplay contributing to the pathogenesis of MS and other neuroinflammatory disorders.

Mitophagy, a selective form of autophagy that targets damaged or dysfunctional mitochondria for degradation and recycling, plays a crucial role in the host’s defense against infections by maintaining mitochondrial quality, regulating immune responses, and preventing excessive inflammation [10]. One of the most well-characterized pathways for mitophagy is the PTEN-induced putative kinase 1 (PINK1)–Parkin Pathway [11]. In this pathway, PINK1 accumulation on damaged mitochondria recruits and activates Parkin (encoded by the *PARK2* gene), leading to the ubiquitination of mitochondrial proteins and the recruitment of autophagy receptors [11]. Notably, mutations in PINK1, Parkin, and other mitophagy-related proteins are linked to early-onset Parkinson’s disease [11].

In a previous study, we investigated *PARK2* knockout (*PARK2^−^^/−^*) mice using electron microscopy and observed mitochondrial abnormalities in the neurons within the substantia nigra of young *PARK2^−^^/−^* mice [12,13]. Specifically, we noted a decrease in mitochondrial matrix density, alterations in the cristae and mitochondrial membrane, as well as an increase in the number of smaller mitochondria in the substantia nigra [12,13]. Additionally, the previous reports have shown that fragmented mitochondria with irregular inner structures build up in aged *PARK2^−^^/−^* mice [14], indicating that defective mitochondria accumulate due to impaired Parkin-mediated mitophagy. Taken together, these findings suggest that Parkin deficiency leads to disruptions in mitochondrial function.

Recent research has demonstrated that PINK1 and Parkin are also associated with mechanisms related to innate and adaptive immunity in the context of neuroinflammation and neurodegeneration. For instance, intestinal infection with Gram-negative bacteria in *PINK1* knockout mice triggers mitochondrial antigen presentation and autoimmune mechanisms, leading to the establishment of cytotoxic mitochondria-specific CD8 T cells in both the periphery and the brain [15]. Furthermore, the absence of the PINK1 and Parkin proteins exacerbates acute inflammation in myelin oligodendrocyte glycoprotein (MOG)-induced experimental autoimmune encephalomyelitis (EAE) in C57BL/6J mice [12,13]. Both proteins have an age-related influence on various subsets of innate and adaptive immune cells in the periphery and CNS at different stages of active EAE progression [12,13]. This age-related impact suggests that the functional roles of PINK1 and Parkin in immune modulation and neuroprotection may vary over the lifespan, affecting the severity and progression of neuroinflammatory conditions such as MS and the other neurodegenerative diseases.

To investigate the relationship between altered mitophagy, EBV, and neuroinflammation, we analyzed the impact of immunization with a recently discovered peptide from the EBV nuclear antigen EBNA1 that cross-reacts with the glial cell adhesion protein (GlialCAM) in the CNS [16]. This study was conducted in both *PARK2* knockout (*PARK2^−^^/−^*) and wild-type mice, and the results were compared with those from immunization using the MOG–EAE model in the same mice. Our study demonstrates that alterations in mitochondria-mediated immune responses lead to increased inflammation due to the EBV antigenic component. This suggests that EBV peptides might exacerbate neuroinflammation through molecular mimicry and mitochondrial dysfunction, highlighting the potential for targeted therapeutic strategies that address these specific immune and mitochondrial pathways in neuroinflammatory diseases such as MS.

## 2. Results

### 2.1. Experimental Autoimmune Encephalomyelitis Progression and Immunization Response in PARK2^−/−^ and Wild-Type Mice

**Figure 1:**
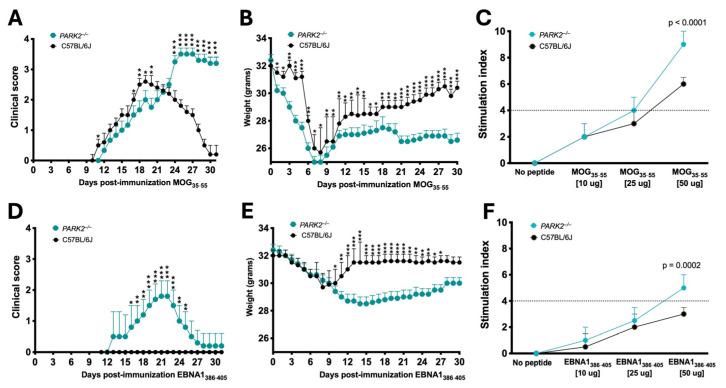
Comparison of disease progression after EBNA1_386–405_ or myelin oligodendrocyte glycoprotein (MOG)_35–55_ immunization between *PARK2*^−/−^ and C57BL/6J mice. Clinical scores and body weights in mice induced by active EAE (**A**,**B**) or in mice immunized with the EBNA1_386–405_ peptide (**D**,**E**). Combined results from three independent experiments, with 20 mice per group. T-cell proliferative response to MOG_35–55_ peptide (**C**) or to EBNA1_386–405_ peptide (**F**). The data show one experiment with 5 mice per group of three independent experiments. Statistical analyses are performed by ANOVA. * *p* < 0.05; ** *p* < 0.01; *** *p* < 0.001.

Firstly, we compared the clinical progression of EAE between the middle-aged *PARK2*^−/−^ and wild-type mice. Our findings confirmed that the absence of *PARK2*^−/−^ significantly exacerbated the severity of EAE, consistent with previous observations in young mice [12]. The peak disease score was significantly higher in *PARK2*^−/−^ mice (3.6 ± 0.5) compared to wild-type controls (3.0 ± 0.5) (*p* < 0.0005) (Figure 1A). Additionally, *PARK2*^−/−^ mice demonstrated delayed recovery 30 d post-immunization. Both groups exhibited similar incidence, mortality, and disease onset (Table 1). All mice exhibited a significant reduction in body weight during the first 10 d after immunization, with the weight loss remaining significant in *PARK2*^−/−^ mice up to 30 d post-immunization (Figure 1B).

Next, we analyzed the effect of EBNA1_386−405_ immunization, which revealed notable differences in the clinical scores between the *PARK2*^−/−^ and wild-type mice (Figure 1D). Notably, only *PARK2*^−/−^ mice displayed symptoms such as EAE, including limp tail, limb weakness, and occasional partial paralysis of the hind limbs. When comparing the *PARK2*^−/−^ mice immunized with MOG_35−55_ or EBNA1_386−405_, the latter group exhibited a delayed onset (12 ± 1.4 d vs. 16 ± 1.4, *p* < 0.0001), reduced severity, and improved recovery 30 d post-immunization (Table 1). All the wild-type mice immunized with EBNA1_386–405_ even exhibited similar body weight changes during the first 10 d post-immunization compared to the *PARK2*^−/−^ mice (Figure 1E). However, after this period, weight recovery in the wild-type mice was associated with the absence of any neurological signs typical of EAE. Clinical characteristics of all mice are reported in Table 1.

Regarding T cell proliferation during the acute phase, preliminary assays indicated that both MOG_35–55_ and EBNA1_386–405_, at a final concentration of 50 mg/mL, had the strongest stimulatory effect on CD3^+^ T cells, with their percentage in the spleen ranging from a mean of 15 to 30%. In middle-aged *PARK2*^−/−^ mice, stimulation with either MOG_35–55_ (Figure 1C) or EBNA1_386–405_ (Figure 1F) significantly enhanced T lymphocyte proliferation compared to the wild-type mice, with a notably stronger stimulatory effect (*p* < 0.0001 and *p* = 0.0002, respectively).

### 2.2. Differential Peripheral Immune Cell Populations and Cytokine Profiles in PARK2^−/−^ Mice Following EBNA1-Immunization and Experimental Autoimmune Encephalomyelitis Induction

**Figure 2:**
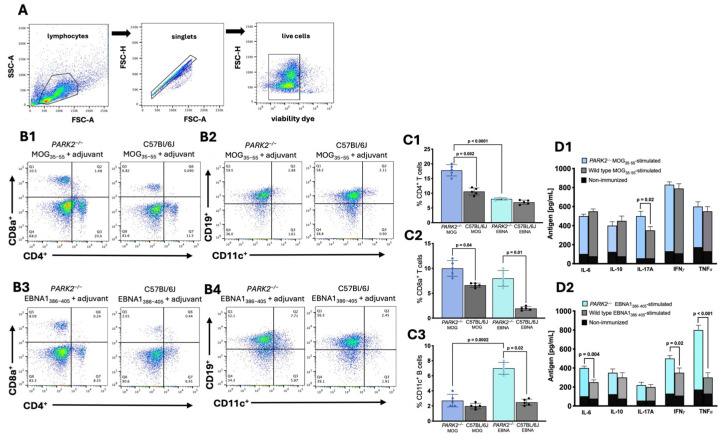
Immune cell populations and cytokine profiles in splenocytes from *PARK2^−/−^* and wild-type mice 20 d post-immunization. The gating strategy for flow cytometry involved using a forward scatter area (FSC-A) versus side scatter area (SSC-A) plot to identify the cells of interest, followed by a forward scatter height (FSC-H) versus forward scatter area (FSC-A) density plot for doublet exclusion, and the assessment of cell viability (**A**). Representative plots showing CD8^+^ and CD4^+^ T cells (**B1**,**B3**) and CD11c^+^ CD19^+^ B cells (**B2**,**B4**), along with corresponding graphs (**C1**–**C3**). Cytokine expression profiles in culture supernatants of splenocytes stimulated with MOG_35–55_, EBNA_386–405_, or not immunized (**D1**,**D2**). The data represent a single experiment with N = 5 mice per group, as part of three independent experiments. Results are presented as mean ± standard deviation, analyzed using Kruskal–Wallis test.

Peripheral cells were isolated from the spleens of the mice 20 d after immunization with either the antigenic peptides or PBS as the placebo control and then analyzed using cytofluorimetry (Figure 2A). No differences were observed in the peripheral immune cell populations between the PBS-immunized *PARK2*^−/−^ mice and the PBS-immunized wild-type mice.

In the middle-aged MOG_35–55_-immunized mice, *PARK2^−/−^* mice exhibited significantly higher levels of both the CD4^+^ (*p* = 0.002) and CD8^+^ (*p* = 0.04) T cells compared to wild-type controls during the acute disease phase of EAE (Figure 2(B1)). The percentage of B cells did not differ between the two groups (Figure 2(B2)).

In the EBNA1_386–405_-immunized mice, the *PARK2^−^^/−^* mice had a higher percentage of CD8^+^ T cells (*p* = 0.01) (Figure 2(B3)) and CD11c^+^ CD19^+^ B cells (*p* = 0.02) (Figure 2(B4)) compared to the wild-type mice, which did not develop any disease symptoms.

When comparing the *PARK2^−/−^* mice immunized with MOG_35–55_ to those immunized with EBNA1_386–405_, there were significant differences in the percentage of CD4^+^ T lymphocytes (*p* < 0.0001) (Figure 2(C1)) but no differences in the CD8^+^ T lymphocytes (Figure 2(C2)). Additionally, the EBNA1_386–405_-immunized mice showed the highest percentage of CD11c^+^ B cells (*p* = 0.0002) (Figure 2(C3)).

Cytokine profiling of the spleen cells from the middle-aged mice immunized with the MOG_35–55_ peptide revealed a significant increase in IL-17 levels in the *PARK2^−/−^* mice compared to the wild-type controls during the acute disease phase of EAE (*p =* 0.02) (Figure 2(D1)). In contrast, the EBNA1_386–405_-immunized *PARK2^−/−^* mice exhibited a significantly higher expression of IL-6 (*p =* 0.004), interferon gamma (IFN-γ) (*p =* 0.02), and particularly that of tumor necrosis factor-α (TNF-α) (*p <* 0.001) compared to wild-type mice (Figure 2(D2)).

### 2.3. Increased Inflammatory Cell Infiltration in Spinal Cords of PARK2^−/−^ Mice Following Immunization with MOG_35–55_ and EBNA11_386–405_ Peptides

**Figure 3:**
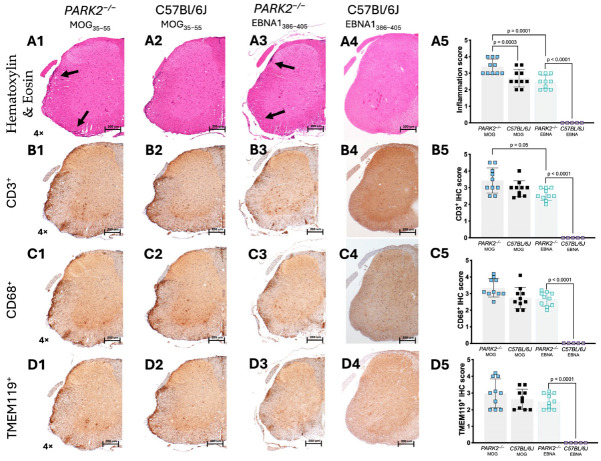
Histological analysis of inflammatory infiltration in the spinal cord of *PARK2^−/−^* and C57BL/6J mice during the acute disease phase. The histological images show sections (scale bar = 200 µm) of the lumbar spinal cord from *PARK2^−/−^* and C57BL/6J wild-type mice after immunization with MOG_35–55_ or EBNA1_386–405_ peptides. These images highlight inflammatory cell infiltration (Hematoxylin and Eosin staining) (**A1**–**A5**), the presence of infiltrated CD3^+^ T cells (**B1**–**B5**) and CD68^+^ macrophages/monocytes (**C1**–**C5**), and the expression of TMEM119^+^ microglia (**D1**–**D5**) in the white matter. For the semi-quantitative analysis of mice, the IHC score was calculated counting the stain-positive cells in two randomly selected sections from ten mice in each group. Arrows indicate areas with inflammatory infiltrates. Data were analyzed using Kruskal–Wallis test.

We analyzed the spinal cords of mice isolated at day 20 during the acute phase using immunohistochemistry. No cell infiltration was detected in the spinal cords of non-immunized control mice. In the spinal cords of the *PARK2^−/−^* mice immunized with MOG_35–55_ to induce EAE, we observed significantly higher inflammatory infiltration (*p* = 0.0003) (Figure 3(A1)) compared to the wild-type controls with EAE (Figure 3(A2)). Following immunization with the EBNA1_386–405_ peptide, the *PARK2^−/−^* mice, which developed a clinical presentation resembling classic EAE, showed typical inflammatory cell infiltration in the spinal cord (Figure 3(A3)). In contrast, no inflammatory cell infiltration was observed in the spinal cord tissue of the wild-type control mice (Figure 3(A4)). When comparing the *PARK2^−/−^* mice immunized with MOG_35–55_ and EBNA11_386–405_ peptides, the former showed a statistically significant decrease in overall inflammation (*p* = 0.001) (Figure 3(A5)).

The inflammatory infiltration was primarily composed of CD3^+^ T cells (Figure 3(B1–B5)), CD68^+^ monocytes (Figure 3(C1–C5)), and TMEM119^+^ microglia (Figure 3(D1–D5)). Immunohistochemistry also revealed a significantly increased number of CD3^+^ T cells in the *PARK2^−/−^* mice immunized with MOG_35–55_, compared to the *PARK2^−/−^* mice immunized with the EBNA1_386–405_ peptide (*p* = 0.05) (Figure 3(B5)).

### 2.4. Different Density of GFAP^+^ Cells in the Hippocampus of PARK2^−/−^ Mice during Inflammation

**Figure 4:**
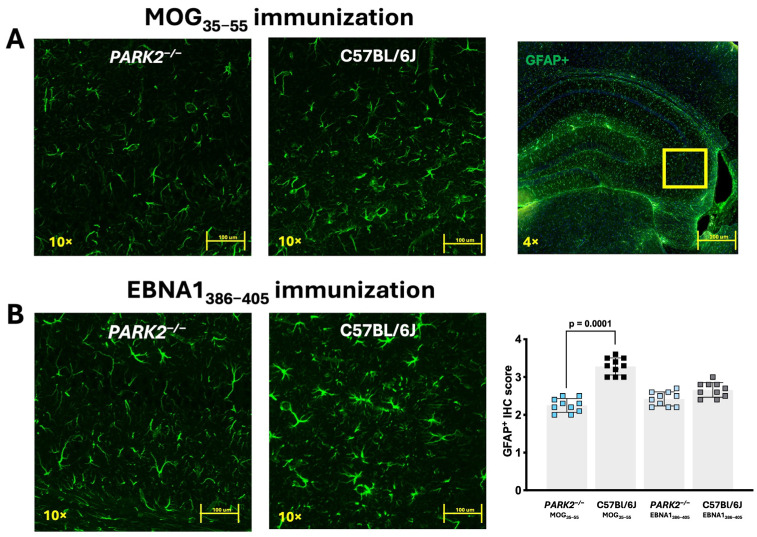
Confocal fluorescence microscopy of GFAP-labeled brain tissue from *PARK2^−/−^* and C57BL/6J mice immunized with MOG_35–55_ or EBNA1_386–405_ peptides during the acute disease phase. The panels show sections (scale bar = 200 µm) of hippocampus from *PARK2^−/−^* and C57BL/6J wild-type mice after immunization with MOG_35–55_ (**A**) or EBNA1_386–405_ peptides (**B**). GFAP^+^ cells were semi-quantitatively analyzed by counting across two randomly selected sections from ten mice in each group. Data were analyzed using Kruskal–Wallis test.

The previous studies have shown different densities of GFAP^+^ cells in the *PARK2^−/−^* mice during inflammation [12]. Based on these findings, we focused our analysis on the hippocampal region of the mice immunized with MOG_35–55_ or EBNA1_386–405_ peptides. GFAP^+^ cells did not significantly differ between the non-immunized *PARK2^−/−^* and C57BL/6J wild-type mice [12]. However, immunofluorescence staining revealed a significant reduction in GFAP^+^ cells across various hippocampal regions in *PARK2^−/−^* mice compared to the wild-type mice during the acute disease phase of EAE (*p* = 0.001) (Figure 4A). In the *PARK2^−/−^* mice immunized with the EBNA1_386–405_ peptide that developed EAE-like symptoms, the number of GFAP^+^ cells was also reduced compared to the C57BL/6J controls (Figure 4B), though this reduction was less pronounced than in the *PARK2^−/−^* mice immunized with MOG_35–55_.

## 3. Discussion

**Figure 5:**
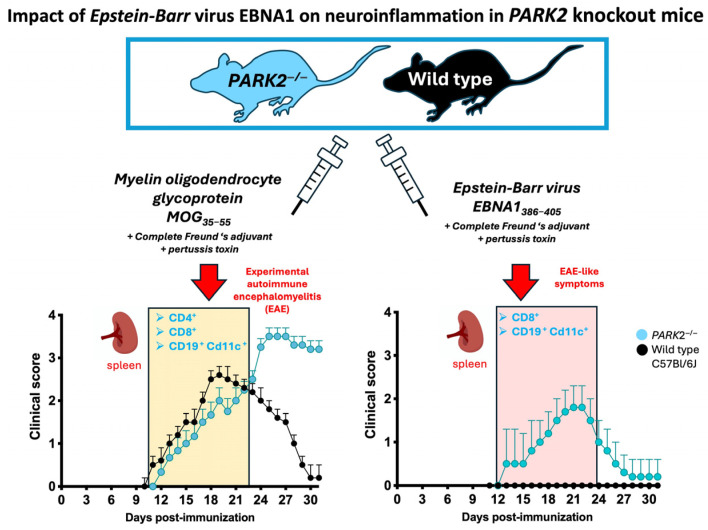
Interplay between Epstein–Barr virus (EBV) and neuroinflammation in experimental autoimmune encephalomyelitis (EAE) in Parkin-deficient (*PARK2*^−/−^*)* mice. Immunization with the EBNA1 peptide (EBNA1_386–405_) triggers a peripheral immune response, characterized by elevated CD8^+^ T and CD11C^+^ B cells. These immune cells, primed by mitochondrial dysfunction and EBV antigen, infiltrate the central nervous system, contributing to neuroinflammation. This model highlights the pathogenic role of EBV in exacerbating neuroinflammation in immuno-mediated diseases, such as multiple sclerosis.

In this study, we confirmed the role of the Parkin protein in modulating peripheral immune cell-mediated immunity during EAE and the role of EBV in neuroinflammation within the context of mitochondrial dysfunction (Figure 5). Specifically, we demonstrated that subcutaneous immunization with the immunogenic peptide EBNA1_385–405_ coadjuvant, along with pertussis toxin administration, can induce symptoms consistent with active MOG–EAE in middle-aged female *PARK2*^−/−^ mice, which exhibit mitophagy associated with mitochondrial dysfunction.

A previous study showed that young *PARK2*^−/−^ mice experienced more severe EAE disease and an earlier onset compared to the wild-type controls, characterized by a high frequency of CD8^+^ T cells in the periphery and brain [12]. This indicates that mitochondrial dysfunction influences the course of MOG–EAE-induced neuroinflammation in mice with a C57Bl/6J genetic background. Another recent study highlighted that EBNA1_386–405_ is involved in high-affinity molecular mimicry with the CNS protein GlialCAM, revealing cross-reactive anti-EBNA1 and anti-GlialCAM antibodies in patients with MS [16]. Additionally, this study showed that young SJL/J mice immunized with the EBNA1_386–405_ peptide a few weeks before inducing active EAE with myelin proteolipid protein (PLP)_139–151_ developed more severe EAE in terms of symptoms, CNS immune cell infiltration, and demyelination [16]. EBNA1_385–405_ also stimulated the secretion of B cell stimulatory T helper cytokines and a strong CD4 response [16].

Our study confirms the encephalitogenic role of EBNA1_386–405_ but with several key differences. Firstly, only *PARK2*^−/−^ mice developed symptoms similar to EAE, not wild-type mice. We did not evaluate the effect of EBNA1_386–405_ before or after MOG–EAE immunization or the effect of a boost. Instead, we assessed the direct immunization effect of EBNA1_386–405_ and compared it to MOG–EAE.

In our model, the *PARK2*^−/−^ mice immunized with EBNA1_386–405_ exhibited a significant percentage of CD8^+^ cells in the periphery compared to the C57Bl/6J wild-type controls that did not develop any symptoms. The Parkin protein encoded by *PARK2* regulates adaptive immunity by repressing mitochondrial antigen presentation [17]. The absence of Parkin can promote the establishment of peripheral mitochondrial antigen-specific T cell populations, which can access the CNS during infections or neuroinflammation [17]. Interestingly, studies investigating anti-EBV immunity in the CNS of MS patients suggest that EBV-specific T cells can gain access to the brain and that altered intrathecal CD8 T cell responses toward EBV could contribute to CNS inflammation and tissue damage [18].

We also observed an increased frequency of CD11C^+^ CD19^+^ B cells in the periphery of the *PARK2*^−/−^ mice immunized with EBNA1_386–405_ compared to the wild-type controls [19]. These cells, primarily memory B cells prone to differentiate into antibody secreting cells [20], continuously expand with age in healthy individuals but show a premature and pronounced accumulation in autoimmune diseases [19]. It has been suggested that these age-related cells are functional mediators of viral-enhanced autoimmunity, as they increase during latent viral infections [19]. The latent form of EBV appears to prime CD11C^+^ CD19^+^ B cells, contributing pathogenically to autoimmune diseases [21,22].

Interestingly, the *PARK2*^−/−^ mice immunized with MOG_35–55_ showed elevated concentrations of the key Th17 cytokine IL-17A in the periphery during the acute phase compared to the wild-type control. In contrast, the *PARK2*^−/−^ mice immunized with EBNA1_386–405_ exhibited lower IL-17A levels but a higher concentration of IL-6, IFN-γ, and TNF-α. Notably, TNF-α was also elevated in the *PARK2*^−/−^ mice immunized with EBNA1_386–405_ compared to those immunized with MOG_35–55._ These findings partially align with previous studies, which have demonstrated that EBNA1_386–405_ stimulated the secretion of IFN-γ, TNF, and IL-12, as well as IL-6 and IL-10, while suppressing IL-17 [16].

Notably, we observed a reduced number of GFAP^+^ cells in the hippocampus of *PARK2*^−/−^ compared to the wild-type mice, regardless of MOG_35–55_ or EBNA1_386–405_ immunization. While GFAP^+^ cells in the hippocampus are primarily associated with astrocytes, they may also include neuronal precursor cells, as embryonic GFAP^+^ cells in young adult mice predominantly differentiate into neurons rather than astrocytes [23]. This reduction in GFAP^+^ cells could potentially compromise the integrity of the blood–brain barrier, increasing the brain’s vulnerability to pathogens, and may also negatively affect neurological recovery.

Another difference is that we selected 9-month-old mice for this study. This age represents a middle-aged mouse, typically extending from 8 to 15 months [24], corresponding approximately to 35 years in humans. The onset of MS usually occurs in women between 20 and 40 years, with an average onset at 35 years. Moreover, a significant proportion of people with MS experience progression independent of relapse activity (PIRA), followed by post-inflammatory neurodegenerative processes over the years. The first attack of PIRA in MS usually occurs at an average age of 32 years [25].

We hypothesize that progressing mitochondrial dysfunction, associated with aging [26] and EBV infection, could be pathological drivers in progressive forms of MS, transiently exacerbating pre-existing symptoms. To test this hypothesis, we require a chronic model of EAE, as our current model induces only a monophasic form of EAE. Additionally, we need to investigate the presence of EBV markers by directly analyzing body fluids from patients with PIRA or progressive forms of MS.

Unbalanced mitochondrial activity is involved in inflammation in several neurological diseases [27]. It is important to note that our studies used *PARK2*^−/−^ mice, which have mutations related to Parkinson’s disease [11]. Despite exhibiting different clinical profiles, common mechanisms related to neurodegeneration, such as mitochondrial dysfunction, are observed in patients with Parkinson’s disease and MS, suggesting converging pathogenic pathways of neurodegeneration. Several cases of co-occurrence of PD after the diagnosis of MS have been reported [28], including a patient with early-onset Parkinson’s disease and a heterozygous PARK2 mutation who, after 8 years, developed primary progressive MS [29]. Moreover, the detection of significantly increased levels of the Parkin protein in the peripheral blood and cerebrospinal fluids of patients with MS [30,31] indicates a potential role for Parkin in the pathogenesis of MS.

Abnormalities in mitochondria are related to a dysfunctional response to infection [8]. Conversely, EBV-encoded proteins during both the latency and lytic infection reduce autophagy, decrease intracellular reactive oxygen species, and modulate mitochondrial function, altering bioenergetics [32]. Clinical and in vitro evidence associate EBV to Parkinson’s disease and the occurrence of parkinsonism [33], with virus-mediated cell cycle dysregulation potentially initiating neurodegenerative processes [34]. However, further studies are needed to clarify the precise role of EBV. For instance, one limitation of our study is that our data were obtained from experiments conducted on female mice. Although the previously published data indicate no differences in the development of EAE between male and female *PARK2*^−/−^ mice [12,13], our findings are restricted to the monophasic EAE model during the effector phase of the disease. Given that in MS, sex differences influence disease pathogenesis, such as men accumulating disability more rapidly than women [35], future research should focus on the recovery phase, utilizing progressive EAE models. In this context, there may be significant differences between male and female mice. Another area for investigation is the impact of mycobacterial components, such as complete Freund’s adjuvant (CFA), on the development of EAE in the *PARK2*^−/−^ mice. The literature indicates that CFA administration can induce mild neuroinflammation [36] and that immunization with mycobacterial components can modulate EAE development [37]. Therefore, it would be interesting to evaluate the effects of CFA alone and other mycobacterial components in our model, particularly in the context of mitochondrial dysfunction.

Despite the areas requiring further clarification, our study demonstrated that alterations in mitochondria-mediated immune responses lead to increased inflammation triggered by the EBV antigenic component. This suggests that EBV peptides may exacerbate neuroinflammation through the mechanism of molecular mimicry and mitochondrial dysfunction. These findings highlight the potential for developing targeted therapeutic strategies that significantly address these immune and mitochondrial pathways in neuroinflammatory diseases like MS.

## 4. Materials and Methods

### 4.1. Generation and Maintenance of PARK2^−/−^ Mice

*PARK2^−^^/−^* middle-aged (9 months old) female mice (N = 20 per group) were generated at Juntendo University [14]. A targeting vector was constructed with 1.5 and 7 kb DNA fragments serving as the 5′ and 3′ homologous sequences, respectively. A negative selection cassette, diphtheria toxin A (DTA), was also included. The linearized targeting vector was transfected into TT2 embryonic stem (ES) cells, and clones were selected in the presence of 300 μg/mL G418 (Merck, Darmstadt, Germany). Screening for homologous recombination was performed by Southern blotting, using a 5′ external probe and a neo-specific probe, confirming the desired recombination in the clones. ES cells from these clones were injected into the C57BL/6J embryos, and chimeric offspring were crossed with C57BL/6J mice to achieve germline transmission, as verified by Southern analysis with the 5′ probe [38]. Heterozygous mice were interbred to produce homozygous knockout mice and wild-type littermate, which were sex- and age-matched as controls. We chose female mice because previous experiments had demonstrated that both knockout and wild-type young (8–12 weeks old) female mice have a high susceptibility to EAE induction [12]. Animals were housed in a pathogen-free facility, maintained under a 12/12 h light/dark cycle. Procedures were approved by the Animal Experimental Committee of the Juntendo University Graduate School of Medicine (approved protocol No. 290238) and conducted in accordance with the NIH and Juntendo University guidelines.

### 4.2. Immunization and Monitoring of PARK2^−/−^ and Wild-Type Mice

Peptides EBNA1_386–405_ (sequence CSQSSSSGSPPRRPPPGRRPF), derived from the EBNA1 protein (UniProt accession number P03211), and MOG_35–55_ (sequence MEVGWYRSPFSRVVHLYRNGK) were chemically synthesized to a purity exceeding 95% by Synpeptide Co., Shanghai, China.

A cohort of *PARK2^−^^/−^* and wild-type mice were subcutaneously immunized with either 200 μg of EBNA1_386–405_ peptide or with 200 μg of the MOG_35–55_ peptide, both emulsified in incomplete Freund’s adjuvant (BD Diagnosis, Fukushima, Japan) supplemented with *Mycobacterium tuberculosis* H37Ra (Difco, Detroit, MI, USA) at a final concentration of 4 mg/mL. Pertussis toxin (200 ng) (Difco) was administrated intraperitoneally to all mice at the time of immunization and 48 h post-immunization to increase blood–brain barrier permeability, facilitating the infiltration of peripheral immune cells into the CNS and amplifying inflammation. All mice were monitored daily, and disease severity was assessed using the following criteria: 0, no clinical signs; 1, flaccid tail; 2, mild hind limb weakness; 3, severe hind limb weakness; 4, hind limb paralysis; and 5, moribund state or death.

### 4.3. Assessment of T Cell Proliferation Using ^3^H-Thymidine Incorporation in MOG_35–55_ and EBNA1_386–405_ Immunized Mice

For the T cell proliferation assay, CD3^+^ T cells were isolated and purified by immunomagnetic positive selection from the spleens of mice that were immunized with either MOG_35–55_ or EBNA1_386–405_ using the MojoSort Mouse CD3 Cell Isolation Kit (BioLegend, San Diego, CA, USA). T cells (4 × 10^5^ cells/well) were cultured for 2 d with 50 μg/mL of the specific peptide. This was carried out in the presence of gamma-irradiated (3000 rad) accessory spleen cells, which were syngeneic to the responding T cells, at a concentration of 1 × 10^6^ cells/mL. Cell proliferation during the last 18 h was measured by determining the radioactivity of the incorporated ^3^H-thymidine (PerkinElmer, Waltham, MA, USA) using a microplate scintillation counter (MicroBeta TriLux, PerkinElmer). The proliferative response was expressed as a stimulation index (counts per minute [cpm] of cells with test peptides/cpm of cells without stimulation) from triplicate determinations.

### 4.4. Cytofluorimetric Analysis of Spleen Cells

For cytofluorimetric analysis, spleen single-cell suspensions (1 × 10^6^ cells) were labeled with live/dead markers (Zombie NIR Fixable Viability Kit, Bio Legend) for 15 min at room temperature, followed by a 10 min preincubation with FcBlock (FcγRII-RIII, BioLegend) on ice. Afterward, the cells were stained on ice for 20 min with fluorochrome-labeled monoclonal antibodies targeting CD4 (GK1.5), CD8a (53-6.7), CD19 (1D3), CD11c (N418), CD5 (53.7.3), Ly6G (1A8), CD11b (M1/70), CD115 (AFS98), and I-A/I-E (M5/114.15.2) [12,13], all purchased from BioLegend, or performed with the appropriate isotype-matched controls (Table 2). Sample acquisition (20,000 events/sample) was performed on a BD FACSCelesta™ (BD Biosciences, Franklin Lakes, NJ, USA), and data analysis was conducted using the FlowJo software version 10.10.0 (FlowJo Company, Ashland, OR, USA).

### 4.5. Cytokine Profiling Following Antigen-Specific T-Cell Stimulation

Spleen cells were isolated from all mice 20 d post-immunization and incubated with either MOG_35–55_ or EBNA1_386–405_ for 48 h, following the previously established protocols [12,13]. Cytokine concentrations in the culture supernatants were then measured using a multi-analyte ELISArray kit (Qiagen, Hilden, Germany), according to the manufacturer’s instructions.

### 4.6. Histology and Immunohistochemical Analysis of Mice Tissues

Mice were transcardially perfused with PBS, followed by 4% paraformaldehyde (PFA, Funakoshi, Tokyo, Japan) in PBS. The brains and spinal cords were then post-fixed in 4% PFA and embedded for sectioning. Paraffin-embedded tissue sections were stained with Hematoxylin/Eosin (Abcam, Tokyo, Japan) to evaluate inflammation, quantified as the percentage of infiltrated area over the total spinal cord sections.

Immunohistochemistry (IHC) and immunofluorescence were performed as previously described [13], utilizing primary antibodies against CD3, CD68, glial fibrillary acidic protein (GFAP), and transmembrane protein 119 (TMEM119), all obtained from Abcam (Table 2). These were followed by an incubation with the appropriate biotinylated or fluorescent secondary antibodies (Invitrogen, Waltham, MA, USA). All samples were processed simultaneously to ensure comparability, and the results were quantified using a previously established semi-quantitative scoring system [13].

### 4.7. Statistical Analysis

Statistical analysis was conducted using GraphPad Prism version 10.2.3 (GraphPad Software, La Jolla, CA, USA). Clinical scores, body weights, and T cell proliferation data were analyzed using two-way analysis of variance (ANOVA). Flow cytometry, cytokine assays, and histological data were assessed with the Kruskal–Wallis non-parametric test followed by Dunn’s post hoc analysis. A *p*-value of less than 0.05 was considered statistically significant.

## Figures and Tables

**Table 1:** Clinical differences between *PARK2*^−/−^ and C57BL/6J wild-type mice immunized with MOG_35–55_ or EBNA1_386–405_.

	MOG_35–55_ Immunization		EBNA1_386–405_ Immunization	
N = 20 × Group	*PARK2^−/−^*	C57BL/6J	*PARK2^−/−^*	C57BL/6J
Incidence	90%	90%	40% *	0%
Mortality	10%	5%	0%	0%
Onset	12 ± 1.4	13 ± 1.5	16 ± 1.4 *	-
Peak	3.6 ± 0.5 *	3.0 ± 0.5	2 ± 1 *	-

* Statistically significant when comparing knockout and wild-type mice with the same immunization.

**Table 2:** List of the primary and secondary antibodies.

Antigen	Technique	Dilution	Catalog No.	Supplier
Primary antibodies				
FITC anti-mouse CD4	Flow Cyt	1:200	100406	BioLegend
Brilliant Violet 421™ anti-mouse CD8a	Flow Cyt	1:100	100738	BioLegend
PerCP/Cyanine5.5 anti-mouse CD19	Flow Cyt	1:100	152406	BioLegend
APC anti-mouse CD11c	Flow Cyt	1:50	117310	BioLegend
PE anti-mouse CD5	Flow Cyt	1:100	100608	BioLegend
Alexa Fluor^®^ 700 anti-mouse Ly-6G	Flow Cyt	1:100	127622	BioLegend
PE/Cyanine7 anti-mouse/human CD11b	Flow Cyt	1:100	101216	BioLegend
APC anti-mouse CD115 (CSF-1R)	Flow Cyt	1:100	135510	BioLegend
Brilliant Violet 510™ anti-mouse I-A/I-E	Flow Cyt	1:100	107636	BioLegend
FITC Rat IgG2b, κ Isotype	Flow Cyt	1:200	400605	BioLegend
Brilliant Violet 421™ Rat IgG2a, κ Isotype	Flow Cyt	1:100	400549	BioLegend
PerCP/Cyanine5.5 Rat IgG2a, κ Isotype	Flow Cyt	1:100	400531	BioLegend
APC Armenian Hamster IgG Isotype	Flow Cyt	1:50	400911	BioLegend
PE Rat IgG2a, κ Isotype	Flow Cyt	1:100	400507	BioLegend
Alexa Fluor^®^ 700 Rat IgG2a, κ Isotype	Flow Cyt	1:100	400528	BioLegend
PE/Cyanine7 Rat IgG2b, κ Isotype	Flow Cyt	1:100	400617	BioLegend
Brilliant Violet 510™ Rat IgG2b, κ Isotype	Flow Cyt	1:100	400645	BioLegend
Anti- CD3	IHC-P	1:100	ab16044	Abcam
Anti-CD68	IHC-P	1:500	ab283654	Abcam
Anti-GFAP	IF	1:1000	ab7260	Abcam
Anti-TMEM119	IHC-P	1:300	ab209064	Abcam
Secondary antibodies				
Goat anti-Rabbit IgG (H+L) Secondary Antibody, Biotin	IHC-P	1:1000	65-6140	Invitrogen
Goat anti-Rabbit IgG (H+L) Alexa Fluor™ 488	IF	1:1000	A-11008	Invitrogen

## Data Availability

The data underlying this article will be shared upon reasonable request to the corresponding author.
